# Quality along the Continuum: A Health Facility Assessment of Intrapartum and Postnatal Care in Ghana

**DOI:** 10.1371/journal.pone.0081089

**Published:** 2013-11-27

**Authors:** Robin C. Nesbitt, Terhi J. Lohela, Alexander Manu, Linda Vesel, Eunice Okyere, Karen Edmond, Seth Owusu-Agyei, Betty R. Kirkwood, Sabine Gabrysch

**Affiliations:** 1 Epidemiology and Biostatistics Unit, Institute of Public Health, Heidelberg University, Heidelberg, Germany; 2 Department of Anaesthesiology and Intensive Care Medicine, Jorvi Hospital, Helsinki University Hospital, Espoo, Finland; 3 Kintampo Health Research Center, Ghana Health Service, Kintampo, Ghana; 4 Maternal & Child Health Intervention Research Group, Faculty of Epidemiology and Population Health, London School of Hygiene and Tropical Medicine, London, United Kingdom; 5 School of Paediatrics and Child Health, University of Western Australia, Subiaco, Australia; 6 Kintampo Health Research Center, Ghana Health Service, Kintampo, Ghana,; Icahn School of Medicine at Mount Sinai, United States of America

## Abstract

**Objective:**

To evaluate quality of routine and emergency intrapartum and postnatal care using a health facility assessment, and to estimate “effective coverage” of skilled attendance in Brong Ahafo, Ghana.

**Methods:**

We conducted an assessment of all 86 health facilities in seven districts in Brong Ahafo. Using performance of key signal functions and the availability of relevant drugs, equipment and trained health professionals, we created composite quality categories in four dimensions: routine delivery care, emergency obstetric care (EmOC), emergency newborn care (EmNC) and non-medical quality. Linking the health facility assessment to surveillance data we estimated “effective coverage” of skilled attendance as the proportion of births in facilities of high quality.

**Findings:**

Delivery care was offered in 64/86 facilities; only 3-13% fulfilled our requirements for the highest quality category in any dimension. Quality was lowest in the emergency care dimensions, with 63% and 58% of facilities categorized as “low” or “substandard” for EmOC and EmNC, respectively. This implies performing less than four EmOC or three EmNC signal functions, and/or employing less than two skilled health professionals, and/or that no health professionals were present during our visit. Routine delivery care was “low” or “substandard” in 39% of facilities, meaning 25/64 facilities performed less than six routine signal functions and/or had less than two skilled health professionals and/or less than one midwife. While 68% of births were in health facilities, only 18% were in facilities with “high” or “highest” quality in all dimensions.

**Conclusion:**

Our comprehensive facility assessment showed that quality of routine and emergency intrapartum and postnatal care was generally low in the study region. While coverage with facility delivery was 68%, we estimated “effective coverage” of skilled attendance at 18%, thus revealing a large “quality gap.” Effective coverage could be a meaningful indicator of progress towards reducing maternal and newborn mortality.

## Introduction

Globally, over 270,000 maternal deaths, 3.3 million neonatal deaths and 2.6 million third trimester stillbirths occur annually [1-4]. To reduce this burden, the World Health Organization (WHO) calls for “skilled care during pregnancy, childbirth and the immediate postnatal period” [5]. Childbirth is a particularly critical time [6]: it is estimated that 42% of maternal deaths, 23% of neonatal deaths, and 32% of stillbirths are intrapartum-related [1]. Interventions to reduce the main causes of death are known and many experts believe health-center-based delivery care is the best strategy [7,8].

Achieving high coverage of delivery services is a necessary but insufficient component of this strategy; increased access to poor quality care will not improve maternal and child health, delivery services must also provide good-quality care [9]. However, measurement of quality is difficult for several reasons [9]. Quality is a multi-faceted concept without a universally accepted definition or common operationalization [10,11]. Evaluating quality in maternity care is further complicated by several features: there are at least two recipients of the services (mother and baby), childbirth is a culturally sensitive issue, and most users of maternal health services are well, but serious complications can develop unpredictably [12].

Availability and quality of maternal care have been evaluated using emergency obstetric care (EmOC) signal functions, interventions that treat the main causes of maternal mortality [5]. The recent addition of neonatal resuscitation to these signal functions acknowledges the continuum of care between mother and baby; however availability of neonatal resuscitation alone does not adequately capture a facilities’ capacity to respond to newborn emergencies [5]. Furthermore, the focus on EmOC has been accompanied by a relative neglect of routine or preventive delivery and postnatal functions, despite clear standards of good clinical practice and the potential to prevent complications from arising [13].

To better take the continuum of care between mother and baby and the importance of routine care into account, a recent proposal called for new signal functions to be added to facility assessments to measure the provision of routine delivery and emergency newborn care in addition to EmOC [13]. Gabrysch et al reviewed current facility survey tools and propose a new set of 23 signal functions that incorporate routine intrapartum and postnatal care as well as emergency obstetric and newborn care [13]. Our health facility assessment is the first to put these recommendations into practice. We evaluated the quality of routine and emergency maternal and newborn care and aspects of non-medical quality at all health facilities in seven districts in the Brong Ahafo Region of Ghana using the newly proposed signal functions as well as the well-known EmOC signal functions. We created composite quality categories based on these signal functions and used these results to estimate the proportion of deliveries in facilities offering high quality care as an estimate of “effective coverage” [14] with skilled attendance. 

### Ethics Statement

Ethical approval for the study was obtained from the London School of Hygiene and Tropical Medicine in the UK, and from the Kintampo Health Research Center in Ghana. Written informed consent for the health facility assessment was obtained from health workers before the start of the interview. All women of reproductive age living in the study area provided written informed consent to the use of their surveillance data in the context of the Newhints trial. 

## Methods

The study site is an area under demographic surveillance, where several large field trials have been conducted [15-18], containing seven contiguous districts in the Brong Ahafo region of Ghana (approximately 15,300 km^2^). This rural region is home to over 120,000 women of reproductive age with around 15,000 live births per year, with a pregnancy-related mortality rate estimated at 377 per 100,000 pregnancies [15] and a neonatal mortality rate of 31 deaths per 1000 live births [17]. 

During October and November 2010, we carried out a health facility assessment in all 86 health facilities in the surveillance area; there was no sampling [19]. A physician and a research assistant conducted interviews with the most senior staff member available, in English and if necessary in Twi. Information was collected on facility type and ownership, opening hours, staffing, and intrapartum and postnatal services. We inquired about the availability of relevant drugs, equipment, and elements of infrastructure using a checklist, and observed selected tracer items. We asked specifically about the number of health professionals conducting deliveries, managing obstetric complications, managing sick newborns and trained in newborn resuscitation. We report the median number of health professionals per facility with interquartile ranges, and percentages of facilities performing individual signal functions. 

We evaluated the quality of care in health facilities in the following four dimensions: 1) routine delivery care, including labour and immediate postnatal care, 2) emergency obstetric care (EmOC), 3) emergency newborn care (EmNC), and 4) non-medical quality. Table 1 lists the signal functions and required tracer items for each dimension of care. Our selection of signal functions was based on functions included in other large-scale facility assessments in consultation with local clinicians (for an overview of the signal functions covered in seven existing facility-survey tools, see Gabrysch et al [13]). For routine care, we included nine of the eleven functions recommended by Gabrysch et al [13], and three additional functions (blood pressure measurement, application of eye ointment, and weighing the baby after delivery). We included all existing EmOC signal functions [5], and six of the eight proposed emergency newborn care signal functions. We also evaluated several non-medical aspects as proxies for acceptability of care: whether the facility allowed mothers the choice to have a companion present at delivery and the status of sanitation facilities.

**Table 1:** Signal functions for four quality dimensions with drugs and equipment, and facility performance of functions, n=64.

**Signal Function**			**Corresponding drugs / equipment**	Facility performance n (%)*
**Routine delivery care**				
		1. Monitor labour with partograph	Correctly filled partograph**^*§*^** + clock**^*§*^** + fetoscope	26 (41)
		2. Use measures of infection prevention during delivery	Sink with soap for hand washing**^*§*^** + clean water source	48 (75)
		3. Measure blood pressure	Sphygmomanometer	61 (95)
		4. Controlled cord traction		52 (81)
		5. Injection of oxytocin within 1 minute of delivery	Oxytocin**^*§*^**	51 (80)
		6. Uterine massage		39 (61)
		7. Place baby on mother’s abdomen after delivery		41 (64)
		8. Dry baby immediately after delivery		53 (83)
		9. Apply eye ointment to the baby’s eyes after delivery		40 (63)
		10. Weigh baby after delivery	Weighing scale	62 (97)
		11. Initiate breast feeding within 1 hour after delivery		63 (98)
		12. Delay bathing at least 6 hours after delivery		26 (41)
**Emergency obstetric care (EmOC)**				
	**Basic Functions**			
		1. Parenteral antibiotic	Ampicillin or Gentamicin	37 (58)
		2. Parenteral oxytocin	Oxytocin**^*§*^**	58 (91)
		3. Parenteral anticonvulsant	Diazepam or Magnesium Sulfate**^*§*^**	59 (92)
		4. Manual removal of placenta		52 (81)
		5. Manual removal of retained products of conception		22 (34)
		6. Instrumental delivery^°^		19 (30)
	**Comprehensive Functions**			
		7. Blood transfusion		10 (16)
		8. Cesarean section		9 (14)
**Emergency newborn care (EmNC)**				
	**Basic functions**			
		1. Injectable antibiotics for newborn sepsis	Ampicillin or Gentamicin	20 (31)
		2. Newborn resuscitation with bag and mask	Bag + mask for baby**^*§*^**	51 (80)
		3. Teach mother skin-to-skin or Kangaroo Mother Care for low birth weight babies		59 (92)
		4. Teach mother to express milk and feed with spoon and cup if baby unable to breastfeed	Graduated measuring cup	25 (39)
		5. Dexamethasone to mother for premature labour^°^	Dexamethasone**^*§*^**	5 (8)
	**Comprehensive functions**			
		6. Intravenous fluids for newborns	Intravenous fluids with infusion sets + Small syringes / needles for babies**^*§*^**	12 (19)
**Non-medical aspects**				
		1. Woman can choose to have delivery companion		39 (61)
		2. Patient toilet exists	Toilet available	56 (88)
		3. Patient toilet is clean	Toilet available + seen + clean**^*§*^**	29 (45)
		4. Patient toilet has water for hand washing	Toilet available + seen + water**^*§*^**	18 (28)
		5. Patient toilet has soap for hand washing	Toilet available + seen + soap**^*§*^**	11 (17)

*In routine delivery functions, n(%) refers to facilities “always” performing each function, for emergency functions n (%) refers to facilities reporting function performance. **^*§*^**Observed tracer items. **^*○*^**Function allowed to be missing in (-1) category.

Table 2 presents the criteria for determining the quality level in each of the four dimensions of care. Our categorization is based on a modification of the categorization of EmOC facilities proposed by AMDD [20] and utilized in a study of EmOC facilities in Zambia [21]. The first step was to assign one point for each signal function if the necessary drugs and equipment were reported available, and if the tracer items were seen (as in Table 1). For routine care, functions depended on the reported frequency of performance; a full point required the function to be “always” performed and half a point was given if the function was performed “often” or “sometimes.” For emergency obstetric and newborn care, we estimated theoretical performance, i.e. relying on reported provision, as opposed to counting functions only as present when actual performance could be assessed via records. 

**Table 2:** Categorization of four quality dimensions.

	**Routine delivery care**		**Emergency newborn care**			**Emergency obstetric care**			**Non-medical**
**Quality / Level of Functioning**	**Number of functions (max. 12)**	Staff*	**Number of Functions (max. 6)**	Staff*	**Other**	**Number of Functions (max. 8)**	Staff*	**Other**	**Number of Functions (max. 5)**
**Highest / Comprehensive (-1)^*§*^**	11-12 (all 12 functions, or 11 with any one function missing)	≥ 3 skilled HP ≥ 2 MW	6 (all functions)	≥ 1 HP present ≥ 3 skilled HP ≥ 1 HP trained in neonatal resuscitation	Electricity available	8 (all 8 functions, or 7 with instrumental delivery missing)	≥ 1 Dr present ≥ 1 Dr conducting CS^#^ ≥ 4 skilled HP ≥ 2 skilled MW	Electricity available	5
**High / Basic (-1)^*§*^**	≥ 8 (any)	≥ 3 skilled HP ≥ 1 MW	≥ 5 (all 5 basic functions, or 4 with dexamethasone missing)	≥ 1 HP present ≥ 3 skilled HP	Referral of neonatal complications + vehicle or phone available	≥ 6 (all 6 basic functions, or 5 with instrumental delivery missing)	≥ 1 HP present ≥ 3 skilled HP ≥ 1 skilled MW	Referral of obstetric complications+ vehicle or phone available	≥ 3 (any)
**Intermediate**	≥ 6 (any)	≥ 2 skilled HP ≥ 1 MW	≥ 3 (any)	≥ 1 HP present ≥ 2 skilled HP	Phone available	≥ 4 (any)	≥ 1 HP present ≥ 2 skilled HP ≥ 1 skilled MW	Phone available	≥ 2 (any)
**Low**	≥ 4 (any)	≥ 1 skilled HP	≥2 (any)	≥ 1 HP present ≥ 1 skilled HP	Phone available	≥ 2 (any)	≥ 1 HP present ≥ 1 skilled HP	Phone available	≥ 1 (any)
**Lowest / Substandard**	No requirements		No requirements			No requirements			No requirements

* health professional (HP) includes doctors (Dr), medical assistants, midwives (MW) and nurses. “Skilled” in routine care refers to HP conducting deliveries. “Skilled” in emergency newborn care refers to HP managing sick newborns. “Skilled” in emergency obstetric care refers to HP trained to manage obstetric complications. § For comprehensive and basic EmOC, “(-1)” signifies instrumental delivery was allowed to be missing and for basic EmNC, “(-1)” signifies that dexamethasone was allowed to be missing. **^*#*^**CS = Cesarean section.

Facilities were grouped according to the number of signal functions they performed, the number of trained health professionals working in the facility, and capacity for referral (see Table 2). We had the strictest requirements for the “highest” quality category, requiring almost all functions and human resource capacity for 24 hour service availability (i.e. at least three staff members, assuming 8-hour shifts). For routine care, we allowed “highest” quality facilities to have one point less than maximum on function requirements, i.e. allowing them to lack one function entirely or to perform two functions less than “always.” All emergency obstetric and neonatal signal functions were required for classification as a comprehensive facility, except for instrumental delivery as this is often not routinely taught or performed [22]. For each quality dimension we report median number of points per facility with interquartile ranges, and the percentage of facilities fulfilling our requirements for each quality category. 

Surveillance data on women of child-bearing age in the study area included information on place of delivery [17,23]. We used our quality categorization to estimate “effective coverage” of skilled attendance in the study region, defined as delivery in a facility with “high” or “highest” quality in all four dimensions. This was done in a cohort of live births with known birthplace (n=15,884) occurring between November 2008 and December 2009, during the conduct of the Newhints trial. 

## Results

### Health facilities

We identified 86 health facilities in the study area. Our analysis is restricted to the 64 facilities offering delivery care: Eleven hospitals (one large public regional hospital, four public district hospitals, two private hospitals and four Christian hospitals), ten private maternity homes (managed by the Ghana Registered Midwives Association), 35 public health centers, and eight “clinics” (comprising clinics, health posts, and CHPS compounds). All delivery facilities reported that they provide emergency services i.e. they have a staff member on call 24 hours a day, seven days a week. 

### Staffing

Our definition of a health professional (HP) includes doctors, medical assistants, midwives and nurses. The 64 delivery facilities employed a median of two HPs conducting deliveries (IQR 1-4); 39% of facilities had at least three HPs conducting deliveries (25/64), four facilities had none. There was at least one HP trained to manage obstetric complications in 92% (59/64) of facilities, 30% (19/64) had at least three. The median number of HPs managing obstetric complications was nine at hospitals (IQR 5-12), two at health centers and maternity homes and one at clinics. There was a median of two doctors able to perform emergency cesarean sections per hospital (Range 0-4). In 95% of facilities (61/64) there was at least one HP able to manage sick newborns, 49% had at least three (31/64). In 88% of facilities (56/64) at least one health professional was trained in neonatal resuscitation, 33% had at least three (21/64).

### Signal functions

**Figure 1:**
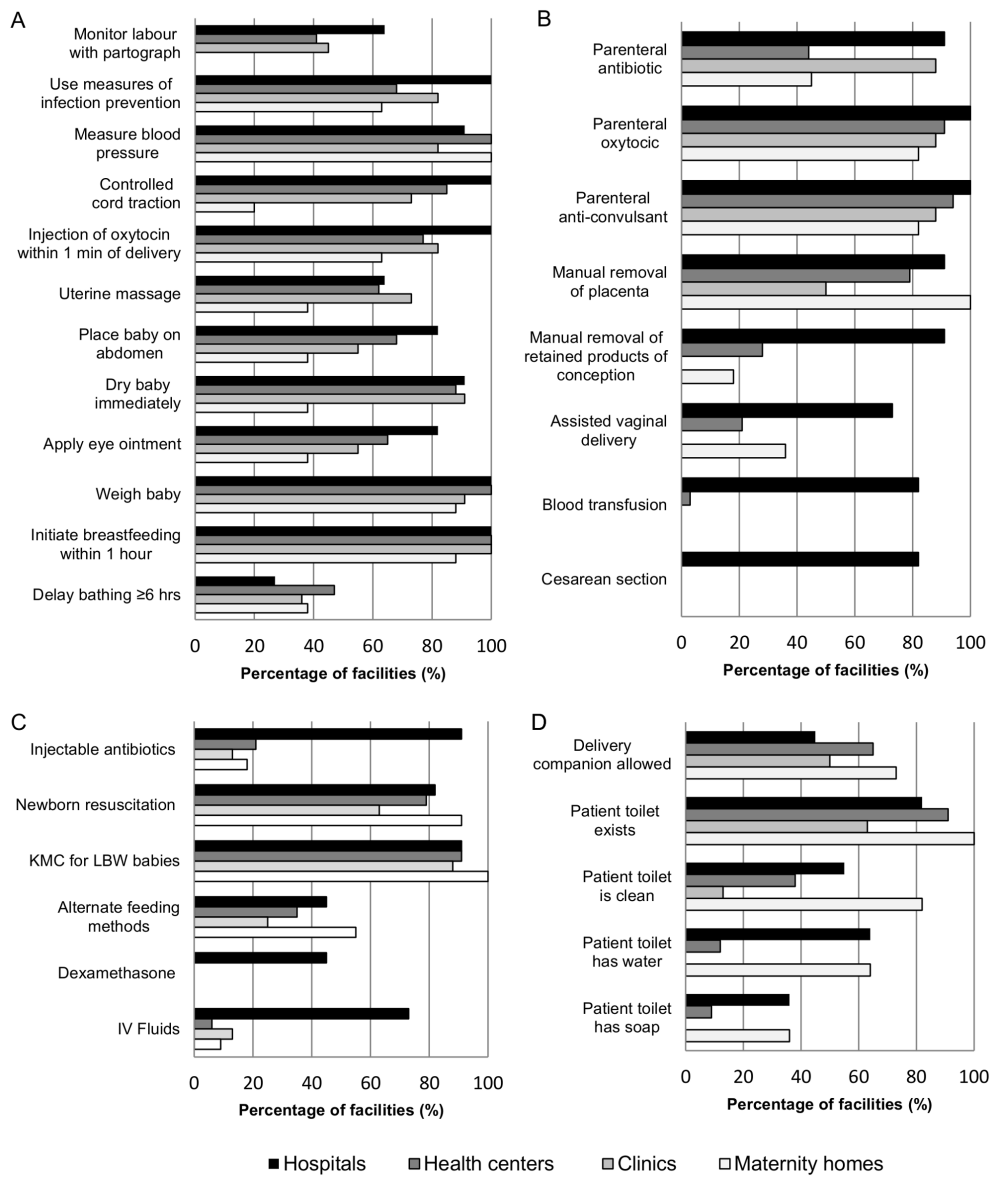
Percentage of facilities performing signal functions by health facility type, n=64 facilities. A. Routine signal functions. Percentage of facilities reporting function “always” performed. B. EmOC signal functions. Percentage of facilities reporting theoretical performance of function. C. EmNC signal functions. Percentage of facilities reporting theoretical performance of function. D. Non-medical aspects. KMC = Kangaroo Mother Care; LBW = low birth weight; IV = intravenous.

For routine delivery care (Table 1, Figure 1A), functions reportedly ‘always’ done in nearly all facilities include monitoring blood pressure, weighing babies and initiating breastfeeding within one hour of delivery. The least frequent routine delivery functions were monitoring labour with a partograph and delaying bathing of the baby for at least six hours after delivery. Although 75% of facilities reported always using partographs, only 41% were able to show correctly completed partographs and had a clock available in the delivery room to help complete the partograph. 

With regards to EmOC functions (Table 1, Figure 1B), most facilities reported provision of injectable anticonvulsants for eclampsia, injectable oxytocics for postpartum hemorrhage, and manual removal of retained placenta, and had the necessary drugs available. The least frequently performed basic EmOC functions were assisted vaginal delivery and manual removal of retained products of conception after abortion complications. Although the majority of hospitals performed all eight EmOC functions, one district hospital was unable to provide injectable antibiotics for sepsis due to a lack of drugs, and two hospitals reported that they could not always provide emergency cesarean sections or blood transfusions.

Teaching mothers skin-to-skin or Kangaroo Mother Care for premature and very small babies was the most commonly reported EmNC function (Table 1, Figure 1C). Performing newborn resuscitation was reported by 88% of facilities and 80% were also able to show a bag and mask during the assessment. Although 98% of facilities reported teaching mothers to express breast milk and feed with a small cup or spoon when newborns were unable to suck, only 39% of facilities also reported having a cup to measure expressed breast milk. Ten of the eleven hospitals as well as one maternity home and one health center reported giving dexamethasone to mothers for preterm deliveries, but only five hospitals had dexamethasone available. 

We also evaluated aspects of non-medical quality as proxies for acceptability and comfort of care, i.e. whether care is “a good experience for the patient” (Table 1, Figure 1D) [9]. More than half of facilities allowed women to choose to have a companion in the delivery room. While most facilities provided a patient toilet, less than half had patient toilets rated as “clean”, less than a third also had water for hand-washing, and few provided soap.

### Overall quality of care categorization

**Figure 2:**
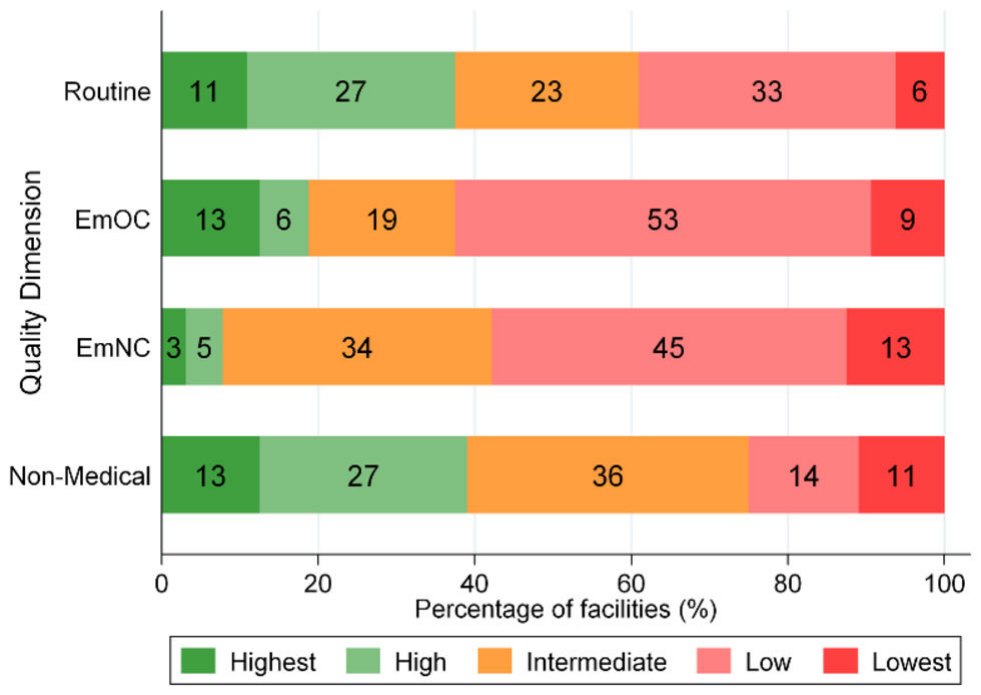
Distribution of facilities across four dimensions of quality, n=64 facilities. Each bar presents the percentage of facilities in each quality level, from “highest” on the left to “lowest” on the right, for each quality dimension. For EmOC and EmNC dimensions, “highest” represents comprehensive (-1) quality; “high” represents basic (-1) and “lowest” represents substandard quality. For comprehensive and basic EmOC, “(-1)” signifies instrumental delivery was allowed to be missing and for basic EmNC, “(-1)” signifies that dexamethasone was allowed to be missing.

Facilities scored a median of 9.5 out of 12 points (IQR 8.25-11) for the performance of routine care signal functions. The median number of skilled health professionals conducting deliveries was 2 (IQR 1-4) and the median number of midwives conducting deliveries was 1 (IQR 1-2). Seven facilities (11%) met the requirements for the “highest quality” category which required ≥11 points and at least three skilled health professionals, at least two of which were midwives: five hospitals, one health center and one maternity home (Figure 2). Another 27% (17/64) of facilities were categorized as “high” quality. Hospitals were all categorized “highest” or “high” quality, while clinics were all “low” or “substandard” quality (see Figure S1 for quality categorization by facility type). 

Less than one fifth of facilities were functioning at EmOC level: Eight hospitals provided comprehensive EmOC, and two hospitals and two health centers provided basic EmOC (Figures 2 & S1). Another one fifth of delivery facilities functioned at an “intermediate” level, and half of all facilities functioned at a “low” level, including one public district hospital that provided only two emergency obstetric functions. Six facilities (9%) were considered “substandard” in terms of EmOC. These facilities either performed less than two EmOC functions, did not employ any health professionals trained to manage obstetric complications, or no health professional was present during our visit. 

The median number of emergency newborn functions performed per facility was 2 out of 6 (IQR 2-3). The median number of health professionals managing sick newborns was 2.5 (IQR 2-4), and median number trained in neonatal resuscitation was 2 (IQR 1-3). Less than 10% of facilities provided comprehensive or basic EmNC, requiring a minimum of four EmNC signal functions, three health professionals managing sick newborns and one health professional present during our visit (Figure 2): Two hospitals fulfilled the requirements for comprehensive EmNC and one hospital and two health centers those for basic EmNC. Seven hospitals functioned at an “intermediate” and one at a “low” EmNC level. This was primarily due to a lack of equipment; two hospitals were missing a bag and mask for neonatal resuscitation, three were missing small syringes and needles for babies, and five reported that they did not have cups for measuring expressed milk. More than half of all facilities were categorized as providing “low” or “substandard” EmNC (Figure 2). 

For non-medical quality, the median score was 2 out of 5 (IQR 1.5-3). In total, 13% of facilities were categorized as “highest” non-medical quality, meaning they provided adequate sanitation facilities and allowed mothers to choose to have a companion during delivery; one quarter were considered “low” or “substandard” quality (Figure 2). Unlike the other facility types, all maternity homes provided at least “intermediate” non-medical quality of care (Figure S1).

### Skilled attendance

**Figure 3:**
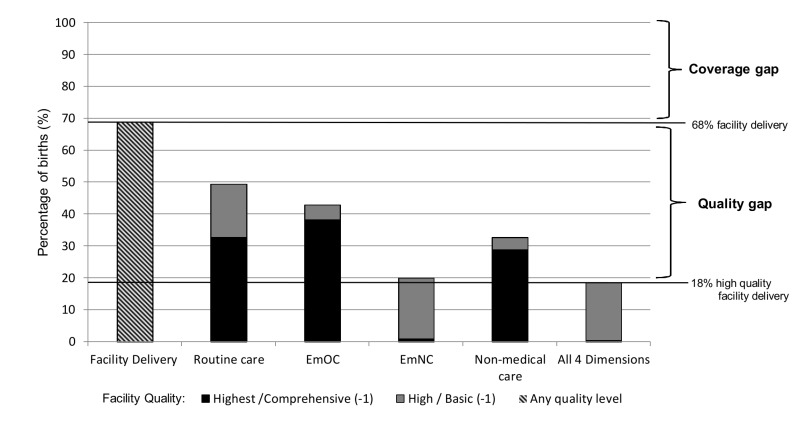
Estimating skilled attendance: percentage of births in facilities with high quality across four dimensions, n=15,884 births. The coverage gap is the difference between current and universal coverage of skilled attendance; with 68% facility delivery in the study region, this gap is estimated at 32%. The quality gap is the difference between coverage with facility delivery (68%), and provision of “effective and client friendly care” i.e. delivery in a facility rated “high” or “highest” on all 4 dimensions of quality (18%). The quality gap was estimated at 50% in the study region (68% - 18%).

There were 16,329 deliveries between November 2008 and December 2009 in the study area, of which 16,168 were live births (99%)[23]. Birthplace was known for 15,884 (98%) of live births, of which 10,782 (68%) were in a health facility. In Brong Ahafo, facility delivery can be used as a proxy for skilled attendance because there are hardly any home deliveries with a skilled provider [24]. In fact, 68% was also the reported national average for skilled attendance in Ghana in 2011 [25]. However, estimates of skilled attendance would be lower if quality of care at facilities was taken into account (Figure 3). Considering the dimensions individually, 49% of deliveries were in facilities with “high” or “highest” quality routine care, 43% with basic or comprehensive EmOC, 20% with “high” or “highest” quality EmNC and 33% with “high” or “highest” non-medical quality. Only 18% of women delivered in a facility rated “high” or “highest” quality on all four dimensions of care simultaneously (fulfilled by three facilities in the study area), and thus can be assumed to have truly received skilled attendance. One facility, a hospital, was in the highest category for all four dimensions, and a small proportion of deliveries occurred at this facility (0.4%). The “coverage gap,” i.e. the difference between current coverage (68% of deliveries in a facility) and universal (100%) coverage, is thus compounded by an even larger “quality gap,” i.e. the difference between coverage with any facility care (68%) and with good quality care (18%). This results in 50% of births in the study area not receiving high quality care although they were in a health facility, representing a large missed opportunity (Figure 3) [26].

## Discussion

We comprehensively assessed quality of care at health facilities in the Brong Ahafo region in Ghana, considering maternal and newborn, routine and emergency care. We used information on performance of signal functions, availability of drugs, equipment and staff necessary to provide 24-hour service, and found that the majority of facilities did not provide high quality care. While 68% of deliveries in the study area were in a health facility, only 18% were in facilities categorized as “high” or “highest” on all four quality dimensions we evaluated. 

Our evaluation showed that facilities that provide a high standard of care in one dimension do not necessarily provide a high standard of care in others. For instance, health facilities providing comprehensive EmOC may not provide the highest quality routine delivery care, and facilities providing high quality obstetric care do not necessarily provide high quality newborn care. In fact, we identified emergency newborn care as the worst-performing dimension in hospitals, with functions and equipment missing even in hospitals providing comprehensive EmOC. These findings underscore the importance of considering the continuum of care for both mother and child in facility assessments [13].

While our substantive findings on facility quality are primarily relevant for Ghana, our study methodology and our multi-dimensional approach could be of broader interest and may serve as an example for other monitoring and evaluation efforts. In the following, we will discuss the rationale for our methodological choices in comparison with alternatives, as well as the implications of our results.

Evaluations of quality of care can take a user perspective through population-based surveys of received services or a provider perspective through facility assessments of available services. Population-based methods depend on patient recall of individual interventions, and the validity of women’s self-report of interventions is variable, with higher validity for location of delivery (hospital vs. health center) as compared to details, such as aspects of active management of the third stage of labour [27]. For routine procedures, such as blood pressure measurement or the application of eye ointment to the newborn after delivery, limited patient recall may lead to underestimation of quality of care. Furthermore, in settings with infrastructural barriers to quality care, such as a lack of drugs, equipment or qualified staff, identifying these problems at their source may be more efficient than asking users [28].

Facility-based assessments of quality employ variations of the following tools: checklists or inventories of infrastructural elements, drugs and equipment; interviews with staff or patients; record reviews; and observation. The scale of assessment ranges from an in-depth evaluation of one facility or ward [29] to a national census of all facilities in a country [30,31]. At the national level, assessments often involve cooperation with several international partners, and can be expensive [30].

The balance between depth and breadth of an assessment, and the choice of tools is determined by both monetary and temporal constraints. As our assessment in Brong Ahafo was done with limited time and budget, and our intention was to include all facilities in the study area for linkage to population data and calculation of geographic accessibility, it was not practical to incorporate observation of care provision, in particular as many facilities in our study area only perform few deliveries. In fact, many of the facilities do not see a sufficient number of patients to perform all signal functions within three months as recommended by the UN and AMDD [5,20]. Actual performance of the signal functions depends on case load, and ‘lack of indication’ was indeed the most common reason why facilities in Ghana did not perform a function, according to AMDD’s national assessment [32]. We therefore relied on reported performance of signal functions, i.e. we used theoretical instead of actual performance to assess emergency care quality.

As we were unable to observe the provision of care, we utilized selected tracer items and incorporated staffing requirements in an attempt to verify interview responses. Lack of tracer items contradicted between 6% (reporting measures of infection prevention but not having a sink with soap) and 58% (reporting provision of dexamethasone for premature labour but not having the drug) of positive responses for a particular function (data not shown), revealing that missing drugs and equipment often limit the quality of care provided. It also suggests that we may have overestimated quality for functions we did not validate with tracer items. However, our results show a low level of quality despite the potential overestimation inherent in our methodology, suggesting that a high level of detail might not yet be necessary when reported performance of functions is already low [33]. Furthermore, this potential overestimation of quality implies that while the facilities we identified as “high” quality may have had deficits we did not detect, the facilities we identified as “low” quality were likely indeed low quality. 

Linking the facility assessment data to population data on facility use, we could show that only one quarter of facility deliveries (18% of 68%) in our study area were in facilities offering “high” or “highest” quality in all four care dimensions. Estimates of “skilled attendance” from population surveys, such as the proportion of deliveries in a facility or with a skilled provider, where quality of care is not considered, are thus far too optimistic, potentially explaining the “paradoxical” disconnect between improving indicators of skilled attendance and persistently high mortality [34]. Efforts to increase facility delivery in Ghana, e.g. through health insurance [35], may reduce the “coverage gap”, however, the “quality gap” between facility delivery and high quality, effective and client-friendly care may remain wide unless efforts are also made to improve quality [26].

## Conclusion

There are several dichotomous elements to consider in maternity care that complicate the operationalization of quality assessments: two recipients (mother and child), two aspects of care (medical and non-medical) and two modes of care (routine and emergency). We advocate that quality assessments of maternal and newborn care acknowledge these and adopt a holistic approach. Our health facility assessment is one example of how this could be done, putting recent recommendations into practice [13]. We found that the overall quality of care in our study region is low; considering all the evaluated dimensions of intrapartum and postnatal care jointly, only three facilities in our study region fulfilled our requirements for “high” or “highest” quality of care.

Wider use of comprehensive facility assessments and their combination with facility utilization data could help move from monitoring coverage (e.g. “skilled attendant at delivery” in Countdown to 2015) to monitoring “effective coverage” of essential maternal and newborn interventions, which is likely to align better with health outcomes [14,34]. It has been suggested that high quality care at birth could even serve as a “litmus test” of “health system quality and performance” in general [26,34].

To reduce the burden of maternal and newborn death, we need to overcome both the “coverage gap” and the “quality gap” [26]. A first step towards improving quality is “to routinely and robustly monitor quality along the continuum of care” [9], and health facility assessments can be an important part of this process [36].

## Supporting Information

Figure S1 **Quality dimensions by facility type in facilities with delivery care, n=64.** A. Routine care quality. B. EmOC. For comprehensive and basic EmOC, “(-1)” signifies instrumental delivery was allowed to be missing. C. Non-medical quality. D. EmNC. For basic EmNC, “(-1)” signifies that dexamethasone was allowed to be missing.
(TIF)
