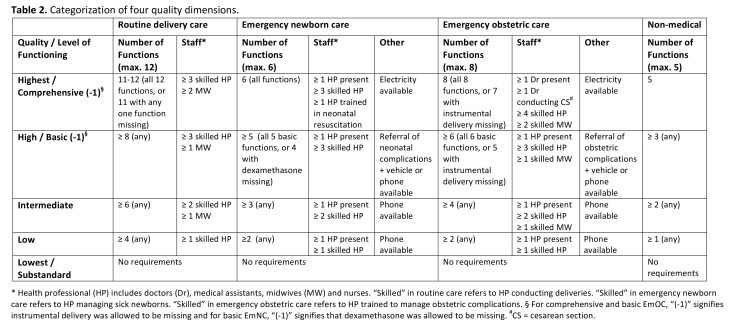# Correction: Quality along the Continuum: A Health Facility Assessment of Intrapartum and Postnatal Care in Ghana

**DOI:** 10.1371/annotation/ac23526c-4e4a-4488-8472-c7de7e3c62f9

**Published:** 2014-01-21

**Authors:** Robin C. Nesbitt, Terhi J. Lohela, Alexander Manu, Linda Vesel, Eunice Okyere, Karen Edmond, Seth Owusu-Agyei, Betty R. Kirkwood, Sabine Gabrysch

Data within the cells of Table 2 should appear on separate lines within the cell. Please see the correct Table 2 here: 

**Figure:**