# Correction: Quality along the Continuum: A Health Facility Assessment of Intrapartum and Postnatal Care in Ghana

**DOI:** 10.1371/journal.pone.0141517

**Published:** 2015-10-20

**Authors:** Robin C. Nesbitt, Terhi J. Lohela, Alexander Manu, Linda Vesel, Eunice Okyere, Karen Edmond, Seth Owusu-Agyei, Betty R. Kirkwood, Sabine Gabrysch

Fig 1 is incorrect. The authors have provided a corrected version here.

**Fig 1:**
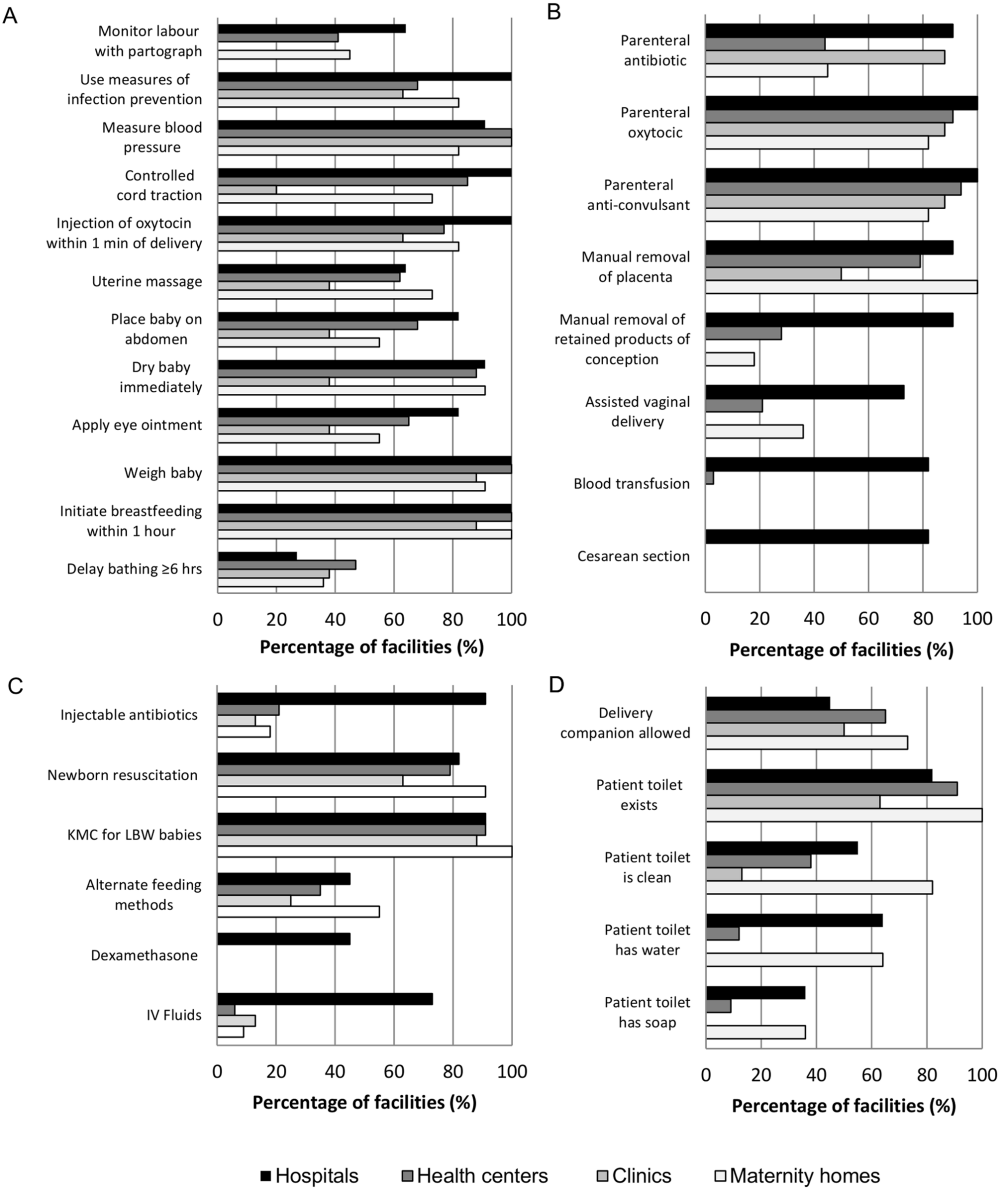
Percentage of facilities performing signal functions by health facility type, n = 64 facilities. A. Routine signal functions. Percentage of facilities reporting function “always” performed. B. EmOC signal functions. Percentage of facilities reporting theoretical performance of function. C. EmNC signal functions. Percentage of facilities reporting theoretical performance of function. D. Non-medical aspects. KMC = Kangaroo Mother Care; LBW = low birth weight; IV = intravenous.

There is an error in the second sentence of the Results section. The correct sentence is: Our analysis is restricted to the 64 facilities offering delivery care: Eleven hospitals (one large public regional hospital, four public district hospitals, two private hospitals and four Christian hospitals), eleven private maternity homes (managed by the Ghana Registered Midwives Association), 34 public health centers, and eight “clinics” (comprising clinics, health posts, and CHPS compounds).
