# Supplementary figures and images for: Quality along the Continuum: A Health Facility Assessment of Intrapartum and Postnatal Care in Ghana
Source: PLoS One. 2013 Nov 27;8(11):e81089. doi: 10.1371/journal.pone.0081089 (PMC3842335; doi:10.1371/journal.pone.0081089)

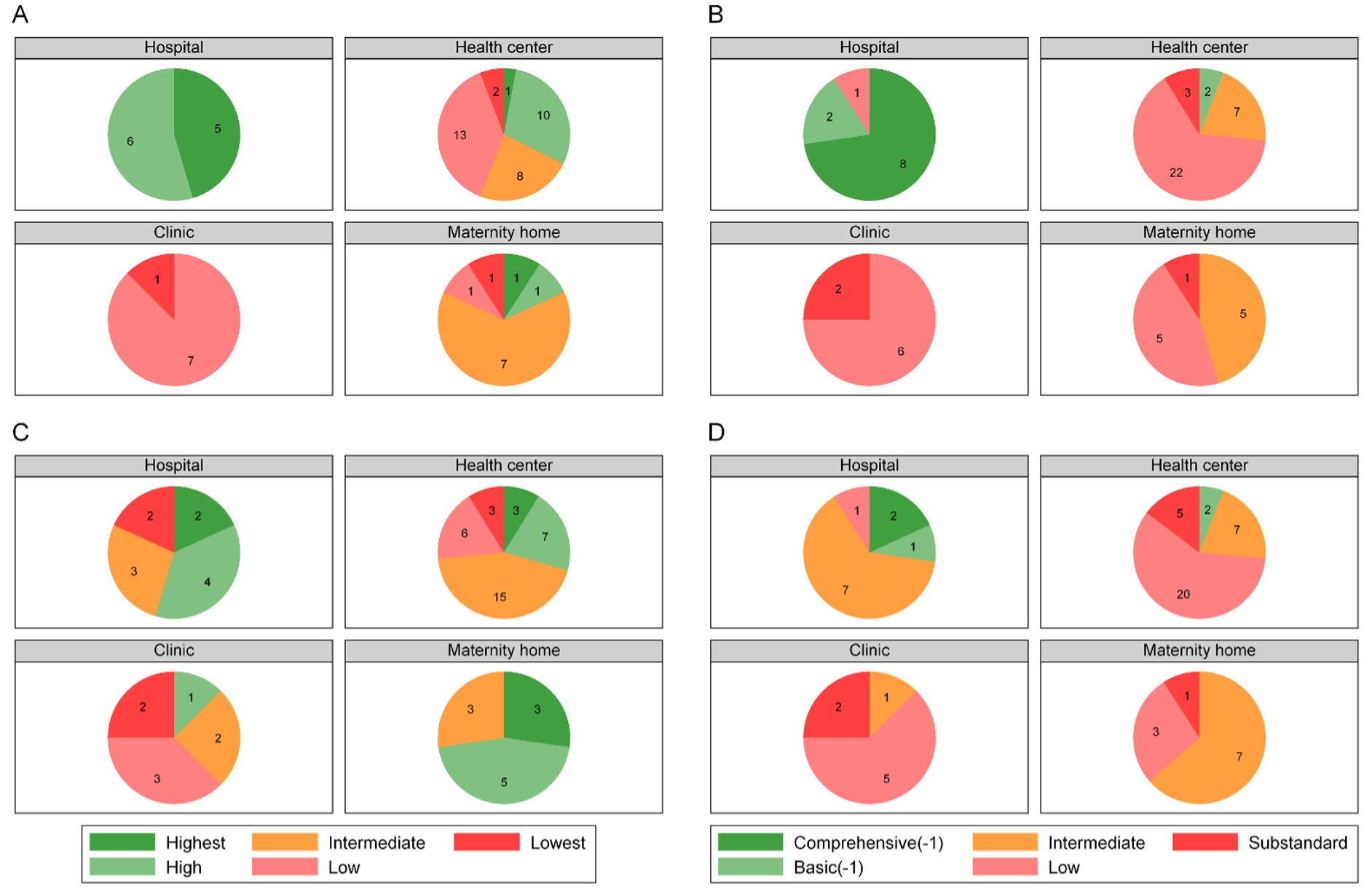

Supplement: Figure S1 — Quality dimensions by facility type in facilities with delivery care, n=64. A. Routine care quality. B. EmOC. For comprehensive and basic EmOC, “(-1)” signifies instrumental delivery was allowed to be missing. C. Non-medical quality. D. EmNC. For basic EmNC, “(-1)” signifies that dexamethasone was allowed to be missing. (TIF) [file pone.0081089.s001.tif]
